# The Benefits of Soft Sensor and Multi-Rate Control for the Implementation of Wireless Networked Control Systems

**DOI:** 10.3390/s141224441

**Published:** 2014-12-18

**Authors:** Raul K. Mansano, Eduardo P. Godoy, Arthur J. V. Porto

**Affiliations:** 1 Group of Automation and Integrated Systems, Univ. Estadual Paulista—UNESP, Av. Três de Março 511, Sorocaba 18087-180, Brazil; E-Mail: rkmansano@yahoo.com.br; 2 Department of Mechanical Engineering, University of São Paulo—USP at São Carlos, Av. Trabalhador São Carlense 400, São Carlos 13566-590, Brazil; E-Mail: ajvporto@sc.usp.br

**Keywords:** soft sensor, mathematical model, wireless sensors, energy efficiency

## Abstract

Recent advances in wireless networking technology and the proliferation of industrial wireless sensors have led to an increasing interest in using wireless networks for closed loop control. The main advantages of Wireless Networked Control Systems (WNCSs) are the reconfigurability, easy commissioning and the possibility of installation in places where cabling is impossible. Despite these advantages, there are two main problems which must be considered for practical implementations of WNCSs. One problem is the sampling period constraint of industrial wireless sensors. This problem is related to the energy cost of the wireless transmission, since the power supply is limited, which precludes the use of these sensors in several closed-loop controls. The other technological concern in WNCS is the energy efficiency of the devices. As the sensors are powered by batteries, the lowest possible consumption is required to extend battery lifetime. As a result, there is a compromise between the sensor sampling period, the sensor battery lifetime and the required control performance for the WNCS. This paper develops a model-based soft sensor to overcome these problems and enable practical implementations of WNCSs. The goal of the soft sensor is generating virtual data allowing an actuation on the process faster than the maximum sampling period available for the wireless sensor. Experimental results have shown the soft sensor is a solution to the sampling period constraint problem of wireless sensors in control applications, enabling the application of industrial wireless sensors in WNCSs. Additionally, our results demonstrated the soft sensor potential for implementing energy efficient WNCS through the battery saving of industrial wireless sensors.

## 1. Introduction

Networked Control Systems (NCSs) are an approach for current applications of distributed control systems using networked architectures for communication and control, in which the controller, plant, sensors and actuators are physically separated and connected through an industrial network [1]. The introduction of networked communication systems can improve the efficiency, flexibility and reliability of these integrated applications through reduced wiring and distributed intelligence with consequent reducing costs in the installation, reconfiguration and maintenance [2]. As result, networked systems technology was quickly applied to accomplish the communication requirements among equipment and systems in control and automation [3]. In NCS approaches, the control signal is sent to the actuator by a message transmitted over the network while the sensor samples the plant output and returns the information to the controller by also transmitting a message over the network.

Recent advances in wireless sensor networking technology have led to development of low cost, low power and multifunctional sensor nodes. These advances have led to an increasing interest in using wireless networks for NCSs [4], resulting in Wireless Networked Control Systems (WNCSs). WNCSs have been attracting many research efforts driven by the growing developments and standardization such as ZigBee [5] and Wi-Fi [6] and for industrial applications such as ISA-100.11a and WirelessHART [7].

According to [8], many interesting properties inherent to wireless networks motivate the development of WNCSs, which have potential to be applied in many large-scale distributed systems, improving their efficiency. Some advantages of WNCS, compared to wired ones, are the ease of installation and maintenance, low cost, and flexibility, as wireless sensors do not require cabling. Therefore, they can operate in a wide range of environments, providing advantages in flexibility and mobility, and are viable in places where cabling is impossible, such as hazardous environments and plants with intrinsic security zones.

Regardless of the networked control advantages, there are effects of nonlinearities and constraints in closed-loop control that may affect the performance and control stability of WNCSs [9]. Some effects are the loss of information when transmitting data, variable communication delays, signal sampling and quantization issues due to information packaging and constraints related to limited bandwidth. Particularly in WNCSs, some problems derive from shared medium conditions, such as unreliability of wireless transmission and interferences [10]. Therefore, the challenge in developing WNCS is to understand the impact of these effects over the system performance and control stability, in order to develop a control strategy that can minimize these effects and reach the control requirements [11].

A fundamental issue and technological concern in WNCS is the energy consumption of wireless devices [12]. Since the devices are powered by batteries, the lowest possible consumption is required to extend battery lifetime and reduce maintenance during operation [13]. However, reducing energy consumption in WNCS may be challenging due to the today's digital control systems requirement for fast updates. Therefore, research about energy-saving solutions for WNCS applications is gaining importance [12,14,15]. In most wireless sensors, the radio is the main energy-consuming subsystem, and then the energy consumption of the wireless sensor is directly related to the data transmission on the wireless network [16]. In this sense, a problem raised in WNCS is the restriction or limitation of the sampling period of industrial wireless sensors, which occurs due to the impracticality of transmitting the sensor data at very fast rates [17]. As a result, there is compromise between the wireless sensor sampling period and its battery lifetime and there may be control applications such as WNCSs in which the wireless sensors cannot be used. Therefore, overcoming this sampling constraint is the first concern to enable the use of these wireless sensors in WNCSs and develop energy-saving solutions for WNC applications.

This paper develops a model-based soft sensor and a multi-rate control strategy for WNCSs, focusing on minimizing energy consumption while guaranteeing stability and control performance. The soft sensor generates virtual data in addition to real sampled data and the multi-rate control allows faster actuation than the minimum sampling period available for the industrial wireless sensor. As a result, the contributions of the paper for practical implementation of WNCS are as follows: overcoming the sampling restriction and increasing battery lifetime of industrial wireless sensors for closed-loop control applications.

This article is organized as follows: after this introduction, Section 2 presents a literature review about energy saving strategies for WNCSs. Section 3 presents the concepts of soft sensor and multi-rate control, which are the basis of the paper. Section 4 describes the experimental architecture and control strategy proposed for enabling the implementation of WNCS and achieving energy saving. Section 5 analyses the experimental results and discusses the benefits of using the soft sensor and multi-rate control for WNCS. Section 6 ends the article, presenting the conclusions of the paper.

## 2. Energy Saving Strategies for WNCS

Typically, wireless sensors include three basic functions: sensing, processing and communication. The sensing subsystem acquires data from a determinate phenomenon in the environment, the processing subsystem processes locally and stores data, and the communication subsystem transmits data. Since wireless sensors are powered by batteries, the energy resources are limited. However, most applications require a long battery lifetime. Therefore, it is necessary to apply energy conservation strategies in order to increase battery lifetime [18] and reliability [19].

Usually, the energy consumption of the sensing subsystem [20] is negligible, compared to other subsystems. However, in some cases, its consumption can be even higher than the consumption of the radio and, in these cases, an energy-saving strategy must be implemented [21]. The power consumption of the processing subsystem is not comparable to the radio, therefore, many strategies process data locally to avoid transmitting unneeded data. In most cases, the communication subsystem is the main power consumer, even when idle [16]. In this sense, some energy conservation strategies consist in control operation modes of the wireless devices (active, idle and sleep), in order to obtain an adequate trade-off between control performance and energy cost, taking in consideration that transition between operation modes involves energy expenditure and latency [22].

In a general view, some approaches applied to Wireless Sensor Networks aim at reducing power consumption through topology control, taking advantage of the network redundancy to activate only a minimum number of nodes to ensure connectivity, allowing the remaining nodes to sleep and save energy. Hence, power management protocols may be applied to the selected nodes, so they can alternate between sleep and wakeup periods, reducing energy consumption [16]. Power management protocols can be implemented on top of a MAC protocol, so they can be application-driven, or integrated with the MAC protocol, so as to optimize medium access [23]. Other approaches can reduce power consumption by focusing on data management. A review of energy efficient strategies for WSN with a focus on data management and compression is given in [21].

Speaking of energy saving in WNCS, a common approach for energy saving has been on the reduction of the wireless transmissions [14,15]. Adaptive sampling approaches [24] allow nodes to optimize their sleep period, generally by processing information locally and deciding, according to some appropriate parameters, whether the sample data is significant and if it is necessary to transmit the information, e.g., when sampled data differs significantly from the last transmitted value. Nevertheless these approaches are not simple when developing WNCS, as there will be additional issues to consider such as the higher probability of packet losses and the robustness to process disturbances.

For control and energy saving in WNCS, one solution that is gaining attention is the aperiodic control theory, which does not require the periodic transmission of measurements and computation of control inputs [15]. It saves energy and proposes a solution to communication with bandwidth limitation. In aperiodic control theory, the time-triggered classical paradigm in which communications between devices occur at pre-defined sampling instants is replaced with an event-triggered approach [25]. Thus, communications occur only when an event happens such as threshold crossing [26] or some stability or control performance requirement is lost [14]. Data prediction may also be used to predict when an event will occur and schedule the next communication, setting how long the sensor may sleep before waking up to communicate [27]. In [15], three recent aperiodic control techniques (event triggered, self-triggered and hybrid scheme) were proposed and evaluated for WNCSs. According to the paper results, all techniques achieved set-point tracking and disturbance rejection, with closed-loop control performances close to the ones obtained with a traditional periodic paradigm. In addition, by the use of aperiodic control it was possible to reduce the energy consumption of the WNCS. One question about the aperiodic control for WNCS that still remains without proper discussion is the robustness against packet losses occurring when transmitting the event triggered communication. It is expected to impact WNCS performance and stability as the wireless communication introduces non-zero packet errors and packet loss probability caused by the unreliability of the wireless transmissions.

Common strategies that have potential to be applied for energy efficiency in WNCS are the model-based control (MBC) and the model predictive control (MPC). These strategies have been developed in NCS with the purpose of reducing communication over the network [28]. The MBC strategy uses the state of the model for control when no feedback is available and the main focus is on making the WNCS robust against packet losses. The MPC strategy for NCSs was presented in [29]. It represents a more complex approach in which a control algorithm solves an optimal control problem, based on the process current state and reference, over a future prediction horizon. For WNCSs, the MPC strategy have been commonly applied to deal with lost measurements and input/output constraints by the use of predicted future process states when the feedback data is unavailable or there are great delays [30]. This paper is based on the ideas of the MBC and MPC strategies, but focusing on a different application: energy efficiency. A model-based soft sensor, that is an estimation technique, is developed to provide additional data to be used by a multi-rate controller, which enables the use of wireless sensors in WNCSs and increases its battery lifetime.

## 3. Soft Sensor and Multi-Rate Control

In any real system, either an industrial plant or chemical process, in order to monitor or control the activities it is necessary to implement a data acquisition system that provides, whenever desired or when possible, the state of variables of interest. Online methods, although faster due to their nature, require specific instrumentation. This may be sufficiently expensive and, therefore, impractical for implementation, or the required sensor may not even exist. In many applications, the measuring devices are subjected to harsh environments, and failures may still occur. Industrial processes have complex inter-relationship between process variables and this requires the use of techniques for obtaining one or more non-measurable variables, from another measurable [31]. With all the difficulties and requirements of measurement systems, in cases when traditional instrumentation cannot be used, a widely used solution is called soft sensors or virtual sensors. According to [32], the term soft sensor is a combination of software, since the models are usually part of a computer program, and sensors, since they are used to obtain similar information to the physical device.

Some relevant characteristics of soft sensors are [31]: they are a low cost alternative to expensive traditional devices; they may work with physical sensors providing useful information for fault detection tasks, making the system more reliable; the can be developed in cheap hardware, such as microcontrollers; they allow the estimation of real-time data, overcoming delays imposed by the slow dynamics of physical sensors, improving the performance of control strategies.

Soft sensor techniques allow estimating the plant behavior through real data and appropriate algorithms, providing additional virtual data to the real capacity of the sensor [33]. Thus, when there is no information from the real sensor, the soft sensor is responsible for generating and providing data to the controller, so that effects of lack of adequate information are minimized. This strategy, wherein the controller execution rate is *N* times greater than the real sensor sampling period is called multi-rate [34]. [35,36] present results of two different control strategies based on multi-rate PID controllers in which the data supplied was twice that of the real sample, *i.e.*, a dual-rate technic. The controller rate is changed according to the NCS time varying delays in [35]. The utilization rate of an Ethernet network used in the NCS is used to modify the controller execution in [36]. Both papers show that using multi-rate controllers is possible to obtain better performance for the NCS compared with conventional (single-rate) systems.

The challenge of using soft sensor techniques is how to generate reliable data to be used by the WNCS controller [32], in order to enable actuation over the system faster than real sampling. A strategy often applied for estimation uses the explicit mathematical model of the plant to determine, from the real information, the future behavior of the plant. Since the model adequately represents the dynamics of the system, it is expected that the virtual information generated is accurate and reliable, and thus, the real control capability of the WNCS can be analyzed.

It could be verified that the major use of soft sensor techniques is to supplement online variable measurements or to predict unavailable variables for process monitoring and control [32]. Usually these techniques focus on enabling control or increasing overall performance. On the other hand, this paper differs from that as it focus on using the soft sensor with a multi-rate controller in order to overcome the sampling period restriction and increasing battery lifetime of industrial wireless sensors in closed loop control applications and WNCS.

## 4. Architecture and Control Strategy

**Figure 1.**
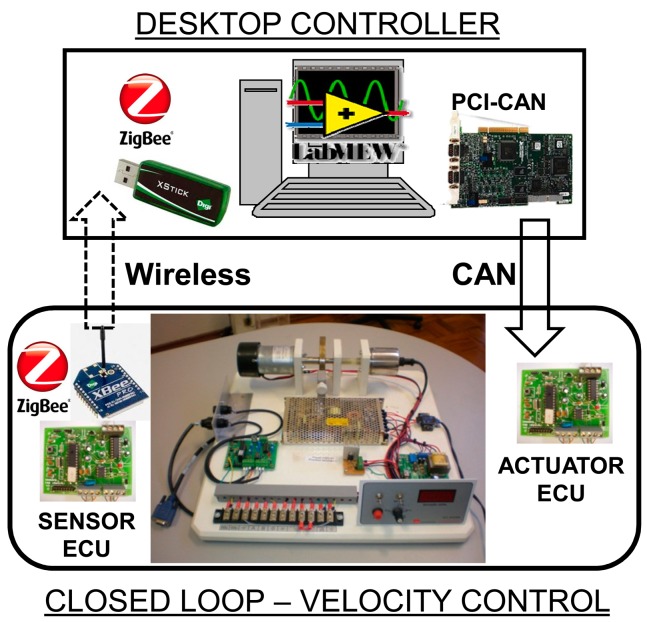
Wireless Networked Control System using CAN and ZigBee.

The testbed used for the experiments in this paper has been developed for WNCS research and experimentation [37]. It is composed of different WNCSs and the networks used are the wired CAN and wireless ZigBee. The experimental setup shown in Figure 1 is related to just one WNCS of the testbed. This setup can adequately reflect a possible industrial application of WNCS.

The defined motor velocity control system has one microcontroller based electronic control unit (ECU) responsible for the sensor data acquisition and transmission of the information in the ZigBee network (using an XBee module from Digi International, Minnetonka, MN, USA) and also responsible for the actuation in the plant and communication with the CAN-based network. A desktop with LabVIEW, a PCI-CAN interface and XStick USB ZigBee device were used for the multi-rate PID control and soft sensor strategy implementation.

Considering the ZigBee network, in the experimental setup the controller is configured as Coordinator and the sensor as a Router or End Device, with point to point communication (no hops on the message transmission). It provides a multicast transmission or the capability of one controller to receive (and control) information from more than one sensor. The time-driven sensor node samples the plant periodically and sends the information to the controller node over the ZigBee network, providing the discretization of the system. Upon receiving a sample, the controller computes a control signal which is sent through the CAN network to the actuator node, where it is subsequently actuated. The threads executing in the controller and actuator nodes are both event-driven, which means that their actions are performed as soon as they receive messages.

**Figure 2.**
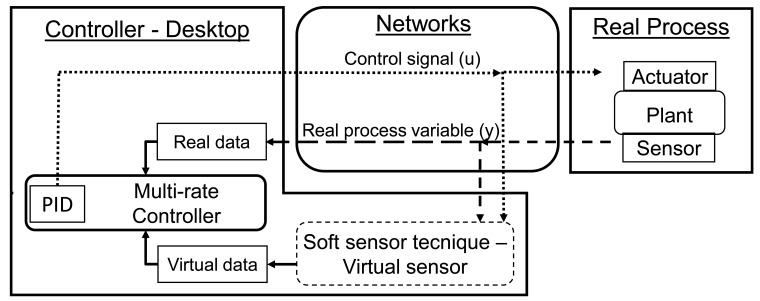
Schematic of the soft sensor strategy with multi-rate control for WNCS.

The block diagram of the soft sensor strategy based on the process mathematical model with multi-rate control is shown in Figure 2. Unlike the strategies described in [28,38] in which the model data was used to compensate packet losses and the model is directly updated, in this paper an online network update scheme is developed and the model data is used mainly for reducing energy consumption. In this network update scheme, the soft sensor running in parallel with the physical process is updated in real-time with the same control signal data transmitted on the network to the actuators (different to the case when the control signal update is done directly without considering the network transmission). The advantage is that the effects of the network delays (in the transmission of the control signal) and the system discretization (the sensor sampling period) on the real process are considered in the mathematical model, providing more reliable virtual data.

In accordance with the Figure 2, the multi-rate PID controller of the WNCS uses virtual data obtained from the soft sensor based on the process model to calculate additional control actions to be sent to the actuator. In this way the actuation on the process is faster than the real sensor sampling, thus overcoming the problem of limitations (or constraints) in the sampling period of industrial wireless sensors. For example, one WNCS with a wireless sensor with a maximum sampling rate of 1 s, with the application of the soft sensor technique and multi-rate control could operate with a closed loop cycle time of 200 ms, in which would have four virtual (soft sensor) samplings for each real sampling of the wireless sensor.

**Figure 3.**
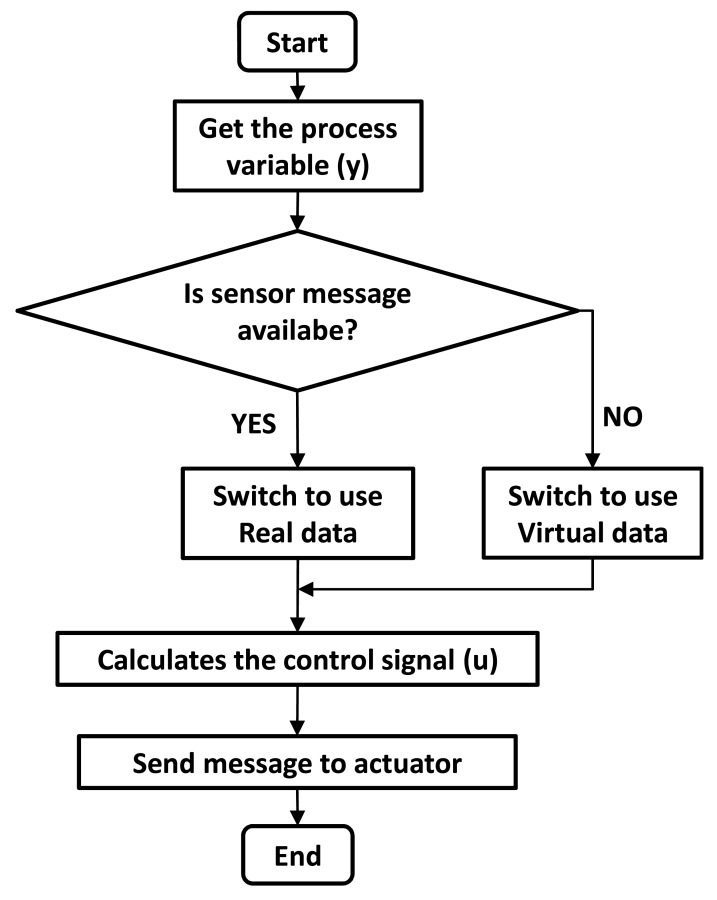
Flowchart of the model-based multi-rate control strategy.

The principle of operation of the soft sensor strategy is detailed in the flowchart shown in Figure 3. Analyzing the flowchart, if the sensor message (process variable) is received by the controller, the real data is used to calculate the control signal to be sent to the actuator. When there is no message transmission from the wireless sensor, the controller uses online updated data obtained from the soft sensor running in parallel with the WNCS. As the soft sensor also receives the control signal sent to the real process, the model output is used as the process virtual data. With this virtual data estimated from the soft sensor (virtual process), the controller calculates the control signal to be sent to the actuator, overcoming the lack of messages from the wireless sensor and guaranteeing the determinism of the closed loop control. The multi-rate control strategy uses an internal PID algorithm to compute the required control signal. The feedback error is obtained by the difference between the setpoint and the process variable, which can be the real data or the virtual data.

As presented in Figure 1, the process to be controlled in this paper is given by a DC motor. The mathematical model of the WNCS for DC motor velocity control used for implementation of the soft sensor is given by Equation (1). This model was experimentally identified using the scheme proposed for WNCS in [38]:

(1) $$\frac{w_{m}}{V}\left(s\right)=\frac{0.0845}{0.006.s+0.008144}.e^{-0.51s}$$

In this model, *w_m_* is the motor angular velocity (soft sensor virtual data used by the multi-rate controller) in rad/s and *V* is the DC voltage applied to the motor (control signal calculated by the multi-rate controller) and *s* is the Laplace variable. It is important to stress that this model is not only the DC motor model, as it takes into consideration the effect of the network in the system. Thus, this model represents the dynamics of the whole WNCS for motor velocity control.

## 5. Results and Discussion

In this paper a PID controller developed in [39] was used for the WNCS application. This controller is a discrete time PID derived with the backward derivative approximation with setpoint weighting and reference off, filtering on the derivative part and an Anti-Windup of the integrative part. For the WNCS operation, the following parameters were used: CAN network speed of 250 kbit/s, messages data length of two bytes for all the ECUs, *N* = 10 in the constant filtering, *B* = 1 in the reference weighting constant and *T*_t_ = *T*_i_ for PI controllers. In addition, a Ziegler-Nichols tuned PI controller was used with *K*_p_ = 0.055 and *T*_i_ = 0.65.

As stated previously, the main problem for the application of wireless sensor networks in NCSs is the limitation (or constraint) in the sampling period, mainly imposed by the energy consumption of industrial wireless sensors [4]. This happens because the energy consumption of the wireless sensor is directly related to the data transmission over the wireless network. Generally, industrial wireless sensors are used in monitoring applications, in which the controlled variable do not need to be transmitted at very fast rates [3]. Due to this focus in monitoring applications and the need to provide a long battery lifetime, these wireless sensors allow a maximum sampling of information, depending on the product model, every 4 s, 2 s or 1 s [40], which limit their application in many closed-loop control applications.

Using the velocity control WNCS first the feasibility of using an industrial wireless sensor for control applications was investigated. It is supposed that the industrial wireless sensor corresponds to the WNCS sensor ECU in Figure 1 and its sampling period is set to 3 s, 2 s and 1 s (which are the maximum possible for these devices).

**Figure 4.**
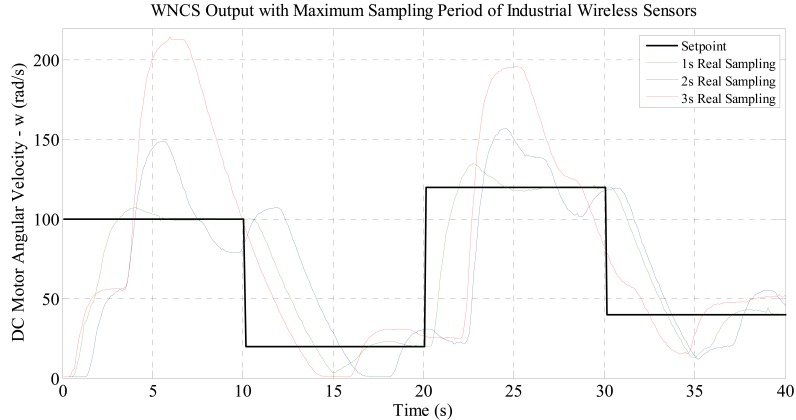
Feasibility of using wireless sensor in closed-loop control applications.

Figure 4 shows the performance obtained for the WNCS for velocity control on a servomechanism configuration. The idea of this experiment is to evaluate if the industrial wireless sensor can be applied in control application such as the WNCS.

The data presented in Figure 4 show unstable and oscillatory response for the slower sampling period (3 s) and an improved response for faster sampling (1 s). However, these results were expected as the maximum sampling period of the wireless sensor is not enough to guarantee the control performance and stability required for the implementation of DC motor WNCS control.

In order to overcome this sampling restriction and enable the use of those wireless sensors in WNCSs, in this paper a soft sensor technique was used with a multi-rate PID controller, allowing actuation on the DC motor *N* times faster than the sampling of the wireless sensor. With the purpose of validating the application of this soft sensor in WNCS, different experiments were defined. In these experiments, the sampling period of the real wireless sensor was maintained constant (1, 2 or 3 s), while the multi-rate period was changed to increase the amount of data generated by the virtual sensor and used by the controller. Thus it would be possible to evaluate the effects of using the soft sensor on the quality of control of the WNCS.

**Figure 5.**
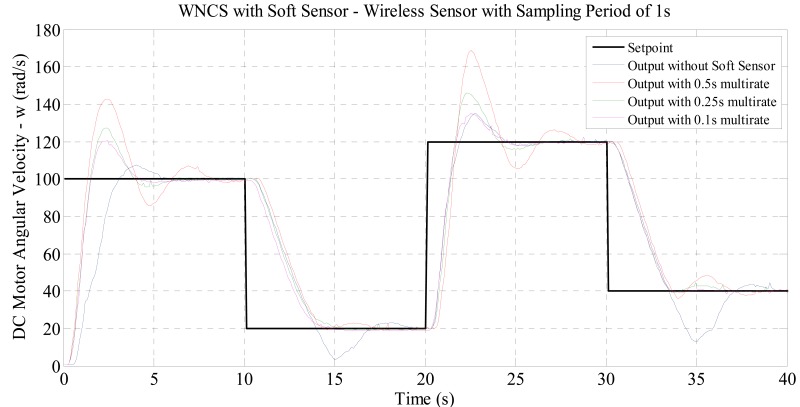
Comparison between the WNCS responses: wireless sensor with real sampling period of 1 s and soft sensor with virtual data sampling periods of 0.5, 0.25 and 0.1 s.

For each sampling period, the motor velocity responses for each experiment were plotted on the same graph, in order to compare the behavior of the system in each case. In addition, two performance indexes were used: IAE (integral of the error) and ITAE (integral of error multiplied by time). These indexes were applied to assess and compare quantitatively the effects of increasing the sampling period of the real wireless sensor and reducing the sampling period of the virtual sensor (or the generation of virtual data) on the performance of the WNCS. For the experiments with WNCS for velocity control set with real sampling period of 1 s, the curves in Figure 5 were obtained.

Analyzing the graph of Figure 5, it is possible to verify that reducing the multi-rate sampling period (virtual data), the response presented a reduction on overshoot and settling time. For these response curves, the performance indexes shown in Table 1 were obtained. The decrease of the indexes values in Table 1 demonstrates that with the soft sensor operating with smaller sampling periods (or higher frequencies) of multi-rate, the resulting control has better performance.

**Figure 6.**
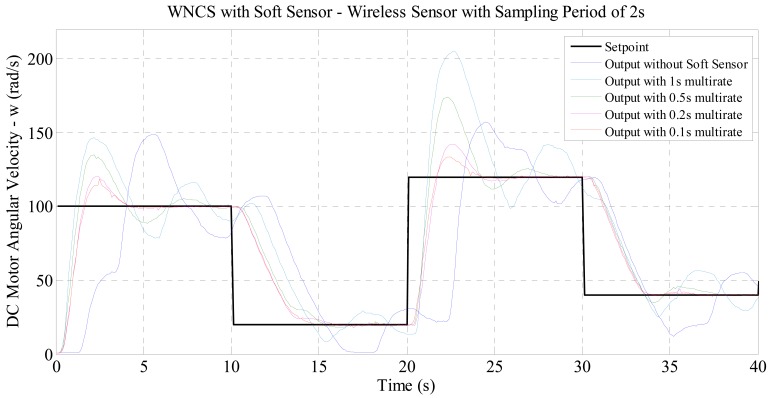
Comparison between the WNCS responses: wireless sensor with real sampling period of 2 s and soft sensor with virtual data sampling period of 1, 0.5, 0.25 and 0.1 s.

For the experiments with WNCS for velocity control set with a real sampling period of 2 s, the curves in Figure 6 were obtained. For the response curves in Figure 6, the performance indexes shown in Table 2 were obtained.

**Figure 7.**
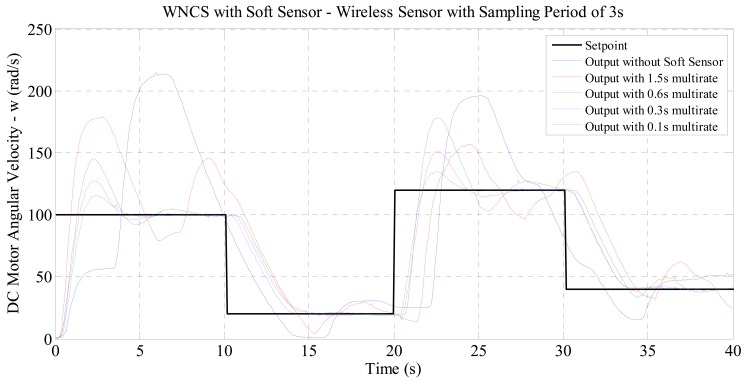
Comparison between the WNCS responses: wireless sensor with real sampling period of 3 s and soft sensor with virtual data sampling period of 1.5, 0.6, 0.3 and 0.1 s.

For the experiments with WNCS for velocity control set with real sampling period of 3 s, the curves in Figure 7 were obtained.

For the response curves in Figure 7, the performance indexes shown in Table 3 were obtained.

**Figure 8.**
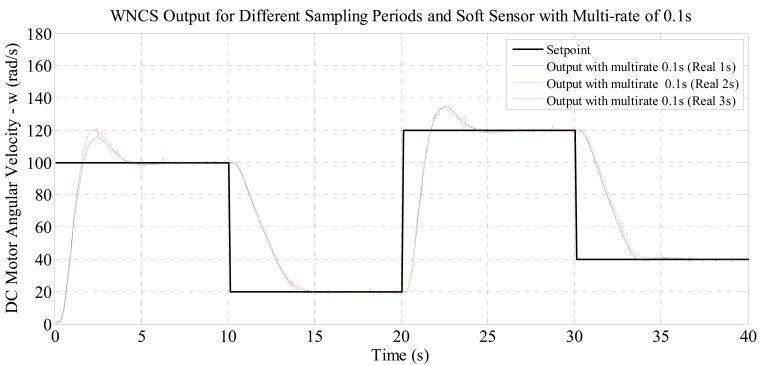
Comparison of control outputs of the WNCS for different sampling periods of the wireless sensor: soft sensor with multi-rate of 0.1 s.

For the experiments with real sampling period of 2 s and 3 s, the graphical results presented in Figures 6 and 7 allow the same observation made for the experiment with real sampling of 1 s. The numerical results in Tables 2 and 3 show that the performance indexes were reduced with the increase of the virtual sampling provided by the soft sensor. The results demonstrate that the soft sensor based on the process model (DC motor identified) developed in this paper enabled the use of industrial wireless sensors in WNCSs. This fact is clearly demonstrated by the graphs of Figures 6 and 7 where by only using the soft sensor, it was possible to satisfactorily control the WNCS according to the configured reference profile. Therefore, it was proved that this soft sensor is an effective solution to the sampling period restriction of wireless sensors in control applications. Additionally, the numerical results of Tables 2 and 3 indicate that the soft sensor originates an improvement in the control performance of the WNCS. Comparing the performance indexes obtained for the three experiments (with real wireless sensor sampling of 1, 2 and 3 s), with the WNCS operating with the soft sensor and multi-rate of 0.1 s, Table 4 and Figure 8 are compiled.

Table 4 and Figure 8 present an important result of this paper. It is possible to verify that for all cases, the values obtained for the performance indexes are very similar (maximum variation of 2.4% between the worst and best case). This result indicates that, even if the real sampling period of the industrial wireless sensor increases (*i.e.*, the time between data transmissions is increased), the model-based soft sensor developed (virtual data generation and multi-rate control) enables the WNCS to satisfactorily control the process. As a result, it is possible to reduce the use of the wireless sensors and thus, the results show great potential for the developed soft sensor technique: saving battery power of the wireless sensors.

It is proven that energy consumption of the wireless sensor is directly related to the transmission of data in the wireless network. This can be verified with the information in Table 5 that compares the expected battery lifetime of industrial wireless sensors according to the sample period. As can be seen in Table 5 [41], by doubling the sampling period of the sensor (from 1 s to 2 s or 2 s to 4 s), it also gives a twofold increase in the expected battery lifetime of the sensor (from 0.6 year to 1.3 years or 1.3 years to 2.2 years). Therefore, it can be said that by reducing the sampling period of a wireless sensor would be possible to extend its battery lifetime. Thus, based on the results of Table 4, it is possible to attest that by using the developed soft sensor it would be possible to optimize the energy cost and save the battery used by the wireless sensor of the WNCS. This conclusion can be verified by the results showing that it was possible to reduce the sampling period of the wireless sensor from 1 s to 3 s without deterioration of control performance for the WNCS.

Finally, by measuring the current consumption of the wireless sensor module during the WNCS operation in the experiments done, it was possible to verify whether the developed soft sensor strategy reduces the energy consumption and, therefore, improves the battery lifetime expectation. Equation (2) shows the power consumption (*P_AVG_*), calculated as the average power during the WNCS operation time (*T*) and using the voltage (*v*) applied and current (*i*) used by the wireless sensor module. The current was measured using a DAQ card with 100 Hz acquisition time:

(2) $$P_{\text{AVG}}=\frac{1}{T}\int_{0}^{\text{T}}\nu (t)i(t)dt$$

Operation profiles were defined for the wireless sensor module, considering the use of sleep mode (common operation mode for some wireless devices in which the radio is turned off while not used) and the real sampling period used. Thus, three different experiments for the WNCS using the soft sensor with multi-rate of 0.1 s were defined:

- 1 s real sampling with soft sensor and without sleep mode;
- 1 s real sampling with soft sensor and with sleep mode;
- 3 s real sampling with soft sensor and without sleep mode;
- 3 s real sampling with soft sensor and with sleep mode.

**Figure 9.**
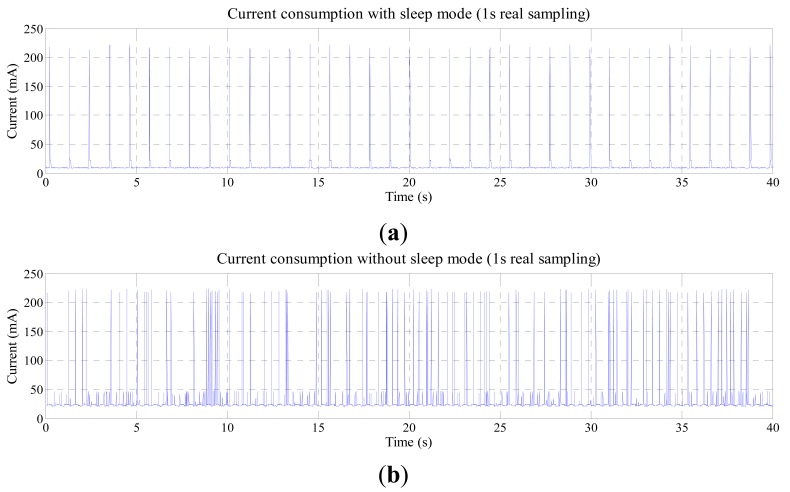
Current consumption during WNCS operation WNCS operation with sampling rate of 1 s (**a**) XBee module using sleep mode; (**b**) XBee module not using sleep mode.

Figure 9 shows the current consumption during operation with 1 s real sampling. Comparing the graphics, when sleep mode is not used, the average current consumption is higher than when using it, and there are more current peaks.

**Figure 10.**
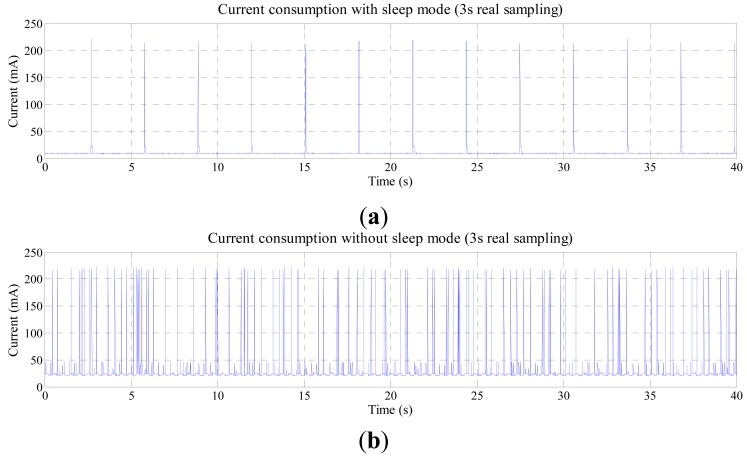
Current consumption during WNCS operation WNCS operation with sampling rate of 3 s (**a**) XBee module using sleep mode; (**b**) XBee module not using sleep mode.

Figure 10 shows the current consumption during operation with 3 s real sampling and the same analysis made for Figure 7 can be made for this case. Table 6 shows the average current and power consumption of the wireless sensor module during the WNCS operation for each profile.

The results show that sleep mode reduced current consumption by 55% for 1 s real sampling rate. Comparing results for 1 s and 3 s of real sampling period, both using sleep mode, the current consumption of the WNCS wireless sensor was reduced by 21%. If the comparison is done against the 1 s real sampling period without sleep mode with the 3 s real sampling period with sleep mode, the current consumption was reduced by 64%. The results, therefore, prove that it is possible to at least maintain the WNCS quality of control and, besides, reduce the wireless sensor power consumption using the soft sensor strategy developed in this paper.

**Figure 11.**
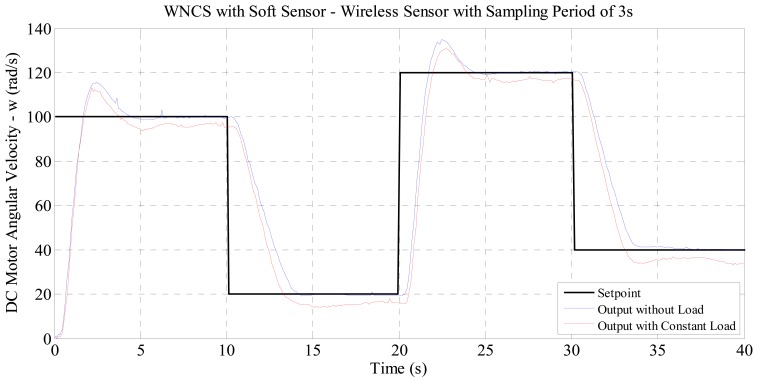
Comparison between the WNCS responses with and without disturbance: wireless sensor with real sampling period of 3 s and soft sensor with multi-rate of 0.1 s.

As the soft sensor technique is based on the process mathematical model and there is no correction or adaptation on the model if the process is under any disturbance, an impact on the WNCS control performance operating with disturbances could be expected. In order to verify that, the performance of the soft sensor technique was compared against an experiment with the motor running with a load, simulating a disturbance. This load is added using a mechanical device that increase the friction between the motor shaft and a screw. The results of this comparison can be seen in Figure 11.

Analyzing the results of Figure 11, the control performance for the case with motor load (that changes the output of the model) presented worse performance than with the identified model. This was expected because if the model used for multi-rate starts to provide virtual data that is not reliable, the control actions calculated by the multi-rate controller will also start to have an undesirable effect on the WNCS.

**Figure 12.**
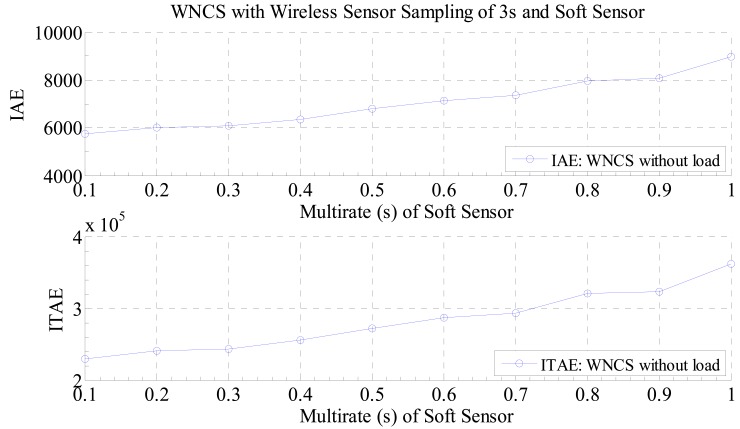
Performance indexes of the WNCS without disturbance: wireless sensor with real sampling period of 3 s and soft sensor with different multi-rate sampling periods.

**Figure 13.**
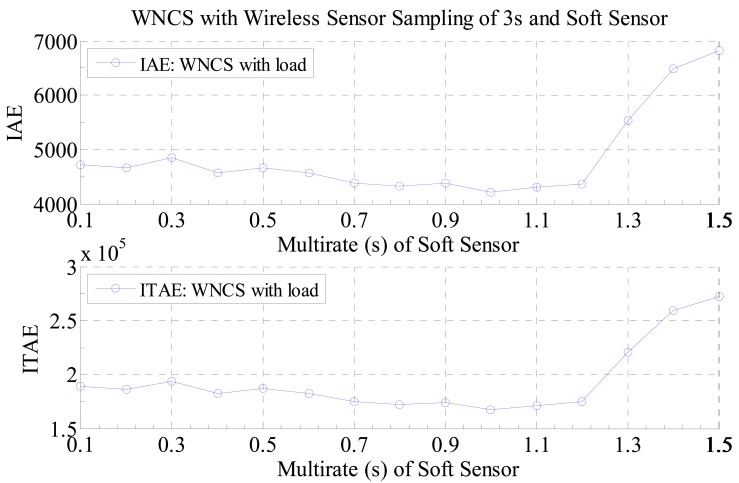
Performance indexes of the WNCS with disturbance: wireless sensor with real sampling period of 3 s and soft sensor with different multi-rate sampling periods.

Therefore, as any real process is not ideal or there always some nonlinearities or disturbances in the control systems, it is important to have a way to evaluate the best multi-rate sampling for a WNCS operating with the developed soft sensor in the presence of disturbances (loads). In that manner, in this paper it as proposed to evaluate this tradeoff by the calculation of the performance indexes Integral of the Absolute Error (IAE) and Integral of Time multiplied by the Absolute Error (ITAE) for different multi-rate sampling periods of the soft sensor for the WNCS operating with disturbance (load on the motor). By plotting these data, it is possible to verify the impact of the multi-rate sampling period on the control performance of the WNCS. In addition, the most important feature of this graph is that it can be used to discover the best multi-rate sampling period that will provide the best control performance for the WNCS. This result can be seen in Figures 12 and 13. For these experiments, the WNCS used the same configurations of the previous experiments: sampling period of 3 s for the real wireless sensor, total running time of 40 s and setpoint profile (different setpoint s in different times).

Analyzing the graphs of Figures 12 and 13, there is a great difference between the curves of the performances indexes when comparing the WNCS running with the soft sensor and with and without disturbance. The curve for the WNCS without disturbance, presented in Figure 12, looks like a growing straight line which means that the point of minimum value for the performance index will be the one with the smallest multi-rate sampling period, so if there is no disturbance on the WNCS (or change on the process output), the virtual data generated by the soft sensor will be reliable and since the multi-rate sampling period of the soft sensor is faster, better is the WNCS control performance.

Nevertheless, if a WNCS with disturbance is considered, the conclusion is different. The curve for the WNCS with disturbance, presented in Figure 13, is different from a straight line and the point of minimum value for the performance index is not achieved for the smallest multi-rate sampling period. As the WNCS is operating with disturbance, the virtual data generated by the soft sensor starts does not fit the real data from the process or it starts to be unreliable. As a consequence, if the multi-rate controller uses all that virtual data to control the WNCS, the control performance is deteriorated. As can be seen in the curve of Figure 13, there is a specific point around the value of 1 s for the multi-rate that provides the best control performance achievable for the WNCS operating under disturbance (load), so using the proposed graph it was possible to obtain the best multi-rate sampling period of the soft sensor that provided the best control performance for the WNCS operating with the soft sensor for saving battery lifetime.

It was possible to verify that the soft sensor and multi-rate control are effective solutions for the implementation of WNCSs. However, the presence of disturbances in the plant under control decreases the efficiency of the proposed strategy as the soft sensor uses a fixed plant mathematical model. In order to improve this result for WNCS plants under disturbances, future work will focus on developing a self-tuning adaptive control strategy for WNCSs. The novelty on this ongoing work is that it will include a multi-rate controller, using data from the model identified online with the adaptive structure, providing energy efficient capability without losing performance because of disturbances on the WNCS plant.

## 6. Conclusions

The results demonstrated, through the comparison of WNCS step response curves and performance indexes, that the soft sensor based on the process model (identified DC motor) allowed the generation of reliable virtual data for the designed multi-rate controller, enabling the use of industrial wireless sensors in WNCSs. Using the virtual data of the soft sensor, it is possible to decrease the cycle time in closed loop (real sensor sampling + soft sensor data) enabling the implementation of control applications previously unavailable with the wireless sensor.

In this way, when the model of the controlled process is known or can be identified, the results indicated that this soft sensor technique is a viable and effective solution to overcome the problem of sampling restriction of wireless sensors in closed loop control applications and in WNCSs.

According to operation data of industrial wireless sensors, by increasing the sensor sampling period, it is possible to reduce proportionally the power consumption, and thus battery lifetime can be increased. The results obtained, in which it was possible to increase the wireless sensor sampling period from 1 s to up 3 s without control performance deterioration, allow concluding that the developed soft sensor technique has great potential for optimizing the energy use of industrial wireless sensor batteries in control applications and in WNCSs.

In order to confirm this technique potential, the current and power consumption of the wireless sensor was measured during the WNCS operation using the soft sensor strategy. Results show that by only increasing the wireless sensor sampling rate, the battery consumption can be reduced by up to 21%. Consequently, in addition to maintaining the WNCS control system performance, the proposed soft sensor strategy is more energy efficient.

Finally by the proposal of a graph comparing the WNCS control performance against multi-rate sampling periods, it was possible to address this important tradeoff of this paper. As a result it could be concluded that when dealing with disturbances, there is a specific and unique multi-rate sampling period, which can be selected to provide the best control performance achievable for the WNCS operating with the soft sensor and consequently save battery lifetime.

## Figures and Tables

**Table 1.** Performance indexes of the WNCS for real sampling of 1 s and soft sensor and multi-rate of 0.5, 0.25 and 0.1 s.

	**Sampling Period**	**IAE**	**ITAE**
Real	2 s	10,968.344	438,747.896

Real + Multi-Rate	0.5 s	7,008.525	280,574.312
	0.25 s	5,896.599	236,066.151
	0.1 s	5,607.121	224,561.200

**Table 2.** Performance indexes of the WNCS for real sampling of 2 s and soft sensor and multi-rate of 1, 0.5, 0.2 and 0.1 s.

	**Sampling Period**	**IAE**	**ITAE**
Real	2 s	10,968.344	438,747.896

Real + Multi-Rate	1 s	9,457.060	378,304.038
	0.5 s	6,969.695	278,908.316
	0.2 s	5,961.231	238,743.079
	0.1 s	5,468.422	218,836.911

**Table 3.** Performance indexes of the WNCS For real sampling of 3 s and soft sensor and multi-rate of 1.5, 0.6, 0.3 and 0.1 s.

	**Sampling Period**	**IAE**	**ITAE**
Real	3 s	13,742.574	550,586.487

Real + Multi-Rate	1.5 s	11,254.125	450,550.900
	0.6 s	7,145.911	285,800.732
	0.3 s	6,082.314	243,793.079
	0.1 s	5,743.923	230,051.518

**Table 4.** Comparison of performance indexes of the WNCS for different sampling periods of the wireless sensor: soft sensor with multi-rate of 0.1 s.

**Real Sampling Period**	**IAE**	**ITAE**
1 s	5,607.121	224,561.200
2 s	5,606.227	226,594.220
3 s	5,743.923	230,051.518

**Table 5.** Estimation of battery lifetime of industrial wireless sensors for different sampling periods [41].

**Industrial Wireless Sensor Emerson Process**	**Sampling Period (s)**	**Expectation of Life (Years)**
Rosemount 3051 Wireless Pressure Transmitter	1	0.6
	2	1.3
	4	2.2

Rosemount 648 Wireless Temperature Transmitter	1	0.9
	2	1.7
	4	2.8

**Table 6.** Comparison of power and current consumption of the wireless sensor module. WNCS using the soft sensor with Multi-rate of 0.1 s.

**Operation Profile**	**Average Current [mA]**	**Average Power [mW]**
Real sampling (1 s) without sleep mode	28.9	94.4
Real sampling (3 s) without sleep mode	28.6	93.6
Real sampling (1 s) with sleep mode	13.0	42.6
Real sampling (3 s) with sleep mode	10.2	33.4

## References

[1] Gupta R.A., Chow M.Y. (2010). Networked control system: Overview and research trends. IEEE Trans. Ind. Electron..

[2] Sauter T., Soucek S., Kastner W., Dietrich D. (2011). The evolution of factory and building automation. IEEE Ind. Electron. Mag..

[3] Galloway B., Hancke G.P. (2013). Introduction to industrial control networks. IEEE Commun. Surv. Tutor..

[4] Fischione C., Park P., di Marco P., Johansson K.H., Sudip K.M. (2011). Design principles of wireless sensor networks protocols for control applications. Wireless Networking Based Control.

[5] Garcia L.R., Lunadei L., Bareiro P., Robla J.I. (2009). A Review of Wireless Sensor Technologies and Applications in Agriculture and Food Industry State of the Art and Current Trends. Sensors.

[6] Paavola M., Leiviska K., Blanes J.S. (2010). Wireless sensor networks in industrial automation. Factory Automation.

[7] Petersen S., Carlsen S. (2011). WirelessHART *versus* ISA100.11a: The format war hits the factory floor. IEEE Ind. Electron. Mag..

[8] Johansson K.H. Motivations, challenges and wireless for control of networked and large scale systems.

[9] Baillieul J., Antsaklis P.J. Control and Communication Challenges in Networked Real-Time Systems.

[10] Naghshtabrizi P., Hespanha J.P., Sudip K.M. (2011). Implementation considerations for wireless networked control systems. Wireless Networking Based Control.

[11] Heemels W.P.M.H., Teel A.R., van de Wouw N., Dragan N. (2010). Networked Control Systems with Communication Constraints: Tradeoffs between Transmission Intervals, Delays and Performance. IEEE Trans. Autom. Control.

[12] Sadi Y., Ergen S.C., Park P. (2014). Minimum Energy Data Transmission for Wireless Networked Control Systems. IEEE Trans. Wirel. Commun..

[13] Lee D. (2008). Energy Harvesting Chip and the Chip Based Power Supply Development for a Wireless Sensor Network. Sensors.

[14] De Castro N.C., Quevedo D.E., Garin F., de Wit C.C. Smart energy-aware sensors for event-based control.

[15] Araujo J., Mazo M., Anta A., Tabuada P., Johansson K.H. (2014). System architectures, protocols and algorithms for aperiodic wireless control systems. IEEE Trans. Ind. Inform..

[16] Anastasi G., Conti M., di Francesco M. (2009). Extending the Lifetime of Wireless Sensor Networks through Adaptive Sleep. IEEE Trans. Ind. Inform..

[17] Åkerberg J., Gidlund M., Björkman M. Future research challenges in wireless sensor and actuator networks targeting industrial automation.

[18] Cacheda R.A., Sánchez A.J.G., Sánchez F.G., Haro J.G., Castaño J.G. (2013). On Maximizing the Lifetime of Wireless Sensor Networks by Optimally Assigning Energy Supplies. Sensors.

[19] Dâmaso A., Rosa N., Maciel P. (2014). Reliability of Wireless Sensor Networks. Sensors.

[20] Casilari E., Garcia J.M.C., Garrido G.C. (2010). Modeling of Current Consumption in 802.15.4 ZigBee Sensor Motes. Sensors.

[21] Razzaque M.A., Dobson S. (2014). Energy-Efficient Sensing in Wireless Sensor Networks Using Compressed Sensing. Sensors.

[22] De Castro N.C., de Wit C.C., Garin F. Energy-aware wireless networked control using radio-mode management.

[23] Razaque A., Elleithy K.M. (2014). Energy-Efficient Boarder Node Medium Access Control Protocol for Wireless Sensor Networks. Sensors.

[24] Ploennigs J., Vasyutynskyy V., Kabitzsch K. (2010). Comparative Study of Energy-Efficient Sampling Approaches for Wireless Control Networks. IEEE Trans. Ind. Inform..

[25] Sánchez J., Guarnes M.A., Dormido S. (2009). On the Application of Different Event-Based Sampling Strategies to the Control of a Simple Industrial Process. Sensors.

[26] Yook J.K., Tilbury D.M., Soparkar N.R. (2002). Trading computation for bandwidth: Reducing communication in distributed control systems using state estimators. IEEE Trans. Control Syst. Technol..

[27] Iino Y., Hatanaka T., Fujita M. Event-predictive control for energy saving of wireless networked control system.

[28] Estrada T., Antsaklis P.A. Performance of Model-Based Networked Control Systems with Discrete-Time Plants.

[29] Liu G.P, Rees D., Chai S.C. Design and practical implementation of networked predictive control systems.

[30] Li H., Shi Y. (2013). Output feedback predictive control for constrained linear systems with intermittent measurements. Syst. Control Lett..

[31] Garcia C., Berni C.C., Oliveira C.E.N. (2008). Hardware/firmware implementation of a soft sensor using an improved version of a fuzzy identification algorithm. ISA Trans..

[32] Fortuna L., Graziani S., Rizzo A., Xibilia M.G. (2007). Soft Sensors for Monitoring and Control of Industrial Processes.

[33] Paulsson D., Gustavsson R., Mandenius C.F. (2014). A Soft Sensor for Bioprocess Control Based on Sequential Filtering of Metabolic Heat Signals. Sensors.

[34] Salt J., Cuenca Á., Palau F., Dormido S. (2014). A Multirate Control Strategy to the Slow Sensors Problem: An Interactive Simulation Tool for Controller Assisted Design. Sensors.

[35] Sala A., Cuenca A., Salt J. (2007). A retunable PID multi-rate controller for a networked control system. Inf. Sci..

[36] Cuenca A., Salt J., Sala A., Piza R. (2011). A delay-dependent dual-rate PID controller over an Ethernet network. IEEE Trans. Ind. Inform..

[37] Godoy E.P., Oliveira T.A., Diniz I.S., Porto A.J.V. A testbed for wireless networked control systems based on CAN and ZigBee.

[38] Godoy E.P., Scorzoni F., Cólon D., Porto A.J.V. Model-based compensation for burst message loss in wireless networked control systems: Experimental results.

[39] Godoy E.P., Porto A.J.V., Inamasu R.Y. Applied Simulation to Evaluate the Quality of Control of Networked Control Systems.

[40] Godoy E.P., Scorzoni F., Porto A.J.V. Evaluating Serial ZigBee Devices for Application in Wireless Networked Control Systems.

[41] Emerson Process Management Power Module Life Estimator http://www3.emersonprocess.com/rosemount/powermodulelifecalculator/default.aspx.

